# Let’s have one more look on the potential power of dynamic susceptibility contrast MRI: time, space, and vascular habitats in locally recurrent high-grade gliomas

**DOI:** 10.1007/s00330-023-10271-5

**Published:** 2023-10-05

**Authors:** Riccardo Pascuzzo, Fabio M. Doniselli, Marco E. M. Moscatelli

**Affiliations:** 1 Neuroradiology Unit, Fondazione IRCCS Istituto Neurologico Carlo Besta, Via Celoria 11, 20133 Milan, Italy; 2 Department of Biomedical Sciences for Health, University of Milan, Milan, Italy

High-grade gliomas (HGGs) including glioblastoma (GBM) are the most frequent malignant brain tumor in adults with an extremely aggressive pathology. Prognosis is dismal with few years of median overall survival (OS), despite the efforts to identify novel therapeutic approaches over the last decades. To date, recommended therapy consists of surgery with maximal safe resection of the nodule with enhancement followed by radiotherapy and chemotherapy [1]. However, recurrence is inevitable, most often occurring locally along the margins of the surgical cavity or, in a minority of cases, far from the initial surgical site [2]. When feasible, a repeated resection should be attempted as correlates with longer survival [3].

Intra-tumor heterogeneity is one of the main pathological characteristics of HGGs and concerns cellular and genetic variability within the tumor as well as in the peritumoral brain zone, and it is considered a key determinant of therapy failure [4]. HGGs are also characterized by abnormal vascular proliferation and irregular angiogenesis, which determines extensive microvascular heterogeneity typically observed in all aggressive tumors. This is particularly relevant in the cerebral tissue because it leads to a disruption of the blood–brain barrier (BBB), interfering with the separation between the intra-vascular and extra-vascular compartment spaces.

Radiologically, intra-tumor heterogeneity translates into characteristic features on conventional MRI such as circinate enhancement, central necrosis, and peripheral T2 hyperintensity both edemigenous and infiltrative [5]. However, a precise recognition of the different tumor components and of their degree of infiltration and aggressiveness is not straightforward with standard imaging sequences. Advanced MRI techniques such as perfusion-weighted imaging add important physiological information to disentangle and quantitatively measure the heterogeneity of the tumoral microenvironment.

The most used perfusion imaging technique in neuro-oncology is dynamic susceptibility contrast (DSC), which is based on the administration of gadolinium followed by the acquisition of rapid or T2-weighted images. As gadolinium passes through the capillary bed, it produces a local magnetic field distortion with a subsequent signal drop that reflects permeability properties of the tissue. For every voxel, the signal intensity is plotted against time and from this curve several parameters can be calculated with their relative maps. Cerebral blood flow (CBF) includes flow information from large vessels, arterioles, capillaries, and venules as well as arteriovenous shunts, which are common in neoplastic tissue. Cerebral blood volume (CBV) represents the volume of blood that flows within the tissue sampled [6]. In HGGs, the disruption of BBB increases the permeability of the capillary bed and gadolinium leaks from vascular space altering normal perfusion parameters. Indeed, CBV and CBF provide valuable information on tumor grading, recurrence, clinical outcome, and response to therapy [7]. Other parameters, related to the time blood takes to flow through the tissue, are less useful in neuro-oncology.

In this issue of *European Radiology*, Wang et al [8] used pre-operative DSC of patients with locally recurrent HGG after maximum safe resection and chemoradiotherapy to investigate whether the spatial recurrence patterns (either within or outside the resection cavity) were associated with prognosis. They also evaluated the possible association between spatial recurrence pattern and DSC parameters measured in distinct tumor compartments (named “vascular habitats”) delineated through the hemodynamic tissue signature (HTS) technique [9]. HTS is a multiparametric clustering method that analyses morphologic and DSC features to define four “vascular habitats”: the high-angiogenic tumor (HAT), which localizes the more perfused area of the enhancing tumor; the low-angiogenic tumor (LAT), corresponding to the area of the enhancing tumor with a lower angiogenic profile; the potentially tumor infiltrated peripheral edema (IPE) surrounding the non-enhancing region; and the vasogenic peripheral edema (VPE) consisting of the remaining edema with the lowest perfusion profile. After image pre-processing, HTS performs the following steps of analysis: (1) two regions of interest (ROIs), the enhancing tumor component and the edema, are segmented using a 3D convolutional neural network based on the morphological images (pre-contrast and post-contrast T1-weighted, T2-weighted, and FLAIR T2-weighted images); (2) the relative CBV and CBF maps are computed from DSC imaging; (3) the enhancing tumor and edema ROIs obtained by the morphological segmentation at step 1 are re-defined using the perfusion information obtained at step 2; (4) a cluster analysis of the perfusion heterogeneity within each of the two re-defined ROIs is performed to detect the four “vascular habitats” described above (HAT and LAT within the enhancing tumor, IPE and VPE within the edema region). Wang et al [8] found that the group with intra-resection cavity recurrence had significantly longer progression-free survival and OS than the group with extra-resection cavity recurrence, and lower DSC perfusion parameters in all vascular habitats. They also aimed to predict the spatial recurrence pattern using preoperative DSC parameters of the vascular habitats and other clinical and radiological factors. To this end, a predictive model was developed using features such as the WHO 2016 grade (III or IV) of the tumor, whether the ventricle was connected to the resection cavity (denoted as “ventricular entry”), and the median relative CBV in the IPE vascular habitat. The proposed model had good classification performances, obtaining 0.833 (95% CI: 0.830–0.836) area under the curve (AUC) after internal validation via bootstrapping. Higher tumor grade, presence of ventricular entry, and higher median relative CBV values in the IPE increased the risk of extra-resection cavity recurrence and, consequently, of poor prognosis.

The use of a reproducible and openly available technique such as HTS to quantitatively measure key parameters of tumor heterogeneity is a strength of this work. However, the proposed model was constructed on a small sample of 69 patients and was only validated internally. Therefore, external validation on a larger cohort of patients is needed to confirm the predictive performance of the selected features. This is especially true given the last update of the WHO central nervous system tumor classification of 2021 has changed the way grading is assigned to gliomas [10].

The results of this study highlight the importance of using advanced MRI techniques in this context. Further studies could verify whether such findings are reproducible not only in groups of patients undergoing the same type of treatment (maximal safe resection and chemo-radiotherapy), but also involving cases of sub-total resection or other therapeutic options such as immunotherapies, which may have a different effect on the risk of extra-resection cavity recurrence. Finally, it may be worth evaluating the utility of more advanced parameters such as radiomic and deep learning features extracted from DSC maps. These methods have been proven to yield more accurate results than standard analyses in several neuro-oncological applications, offering an opportunity to improve the tumor segmentation and prognosis.

## Declarations

### Guarantor

The scientific guarantor of this publication is Riccardo Pascuzzo.

### Statistics and biometry

No statistical methods were necessary for this paper.

### Methodology

• Commentary
